# Gut microbiota profiling for risk stratification of surgical intervention in preterm infants with necrotizing enterocolitis: a retrospective cohort study

**DOI:** 10.3389/fmed.2026.1827128

**Published:** 2026-06-01

**Authors:** Xiaolong Li, Fei Peng

**Affiliations:** Department of General Surgery, Northwest Women and Children’s Hospital, Xi’an, China

**Keywords:** gut microbiota, necrotizing enterocolitis, preterm infants, risk stratification, surgical intervention

## Abstract

**Objective:**

To investigate whether gut microbiota characteristics are associated with surgical intervention status in preterm infants with necrotizing enterocolitis (NEC) and to evaluate their potential discriminatory value for surgical risk stratification.

**Methods:**

This retrospective study included 56 preterm infants with NEC admitted to Northwest Women’s and Children’s Hospital between May 2020 and May 2023, including 33 managed non-surgically and 23 who underwent surgery, as well as 30 preterm infants without NEC as controls. Blood samples were collected to measure prostaglandin E2 (PGE2), interleukin-6 (IL-6), interleukin-10 (IL-10), tumor necrosis factor-α (TNF-α), and C-reactive protein (CRP). Fecal samples were subjected to 16S rRNA gene sequencing to assess microbial diversity and taxonomic composition. Sequencing quality was evaluated using rarefaction analysis, β-diversity differences were tested by PERMANOVA, and differential taxa were further reanalyzed using CLR transformation with FDR correction. Associations between gut microbiota indicators and surgical intervention were analyzed using multivariable logistic regression adjusted for clinically relevant covariates, including gestational age, birth weight, postnatal age at NEC diagnosis, Bell stage, antibiotic exposure, feeding type, length of hospital stay, and probiotic use. Receiver operating characteristic (ROC) curves were used to evaluate discriminatory performance for distinguishing surgical NEC from non-surgical NEC within this retrospective cohort.

**Results:**

No significant differences were observed among the three groups in sex, mode of delivery, 1-min Apgar score, or maternal complications (all *P* > 0.05). In contrast, gestational age was lower in the NEC groups than in controls, and infants in the surgical group had a younger postnatal age at NEC diagnosis, a higher proportion of Bell stage III disease, and more frequent exposure to prolonged antibiotic treatment and formula-predominant feeding. The proportion of meconium-stained amniotic fluid was also significantly higher in the surgical group (47.8% vs. 18.2% and 6.7%, *P* = 0.001). Compared with the non-surgical and control groups, the surgical group showed significantly higher levels of IL-6, IL-10, CRP, and TNF-α (all *P* < 0.001), along with lower microbial diversity as indicated by reduced Chao and Shannon indices (both *P* < 0.001). At the taxonomic level, the surgical group exhibited lower relative abundances of Firmicutes, γ-Proteobacteria, Bifidobacterium, and Lactobacillus, but higher relative abundances of Proteobacteria, Salmonella, and Clostridium (all *P* < 0.001). In multivariable analysis adjusted for gestational age, birth weight, postnatal age at diagnosis, Bell stage, antibiotic exposure, feeding type, length of hospital stay, and probiotic use, Bifidobacterium (adjusted OR 0.63, 95% CI 0.44–0.89), Chao index (adjusted OR 0.72, 95% CI 0.56–0.93), and Shannon index (adjusted OR 0.68, 95% CI 0.49–0.94) remained inversely associated with surgical intervention, whereas Proteobacteria (adjusted OR 1.34, 95% CI 1.08–1.67) and Clostridium (adjusted OR 1.51, 95% CI 1.12–2.04) remained positively associated with surgery (all *P* < 0.05). The combined model incorporating Firmicutes, Proteobacteria, γ-Proteobacteria, and Clostridium achieved an AUC of 0.904 (95% CI 0.823–0.985), with 78.62% sensitivity and 88.54% specificity.

**Conclusion:**

Gut microbiota dysbiosis in preterm infants with NEC was associated with surgical intervention status, and part of these associations remained significant after adjustment for key clinical covariates. A combined microbiota-based model showed potential discriminatory value for surgical risk stratification within this retrospective cohort; however, because groups were defined according to final treatment outcome, these findings should not be interpreted as evidence of true prospective prediction or guidance of surgical timing. These findings require confirmation in larger prospective multicenter studies.

## 1 Introduction

Necrotizing enterocolitis (NEC) is the most common and life-threatening gastrointestinal emergency in preterm infants. Its incidence is inversely related to gestational age, reaching 5–10% in extremely low birth weight infants (<28 weeks of gestation), with associated mortality rates of 20–30%. Beyond acute mortality, survivors often suffer from long-term complications, including short bowel syndrome and neurodevelopmental impairment, imposing substantial burdens on patients, families, and healthcare systems (1–3). Despite considerable advances in perinatal medicine and neonatal intensive care over recent decades, early diagnosis and optimal timing of surgical intervention for NEC remain formidable clinical challenges. Current assessment strategies rely heavily on Bell staging criteria, abdominal radiographic findings, and serum inflammatory markers such as C-reactive protein (CRP) and interleukin-6 (IL-6). However, these indicators lack sufficient specificity and predictive accuracy to determine the optimal surgical window. Consequently, some infants experience delayed intervention, leading to catastrophic outcomes such as intestinal perforation and sepsis, whereas others may undergo unnecessary intestinal resection due to overly aggressive surgical decision-making (4, 5). This clinical dilemma underscores the urgent need for more precise biomarkers to assist clinical risk assessment related to surgical NEC.

In recent years, the relationship between gut microbiota and NEC pathogenesis has become a central focus of research. Accumulating evidence indicates that aberrant intestinal microbial colonization in preterm infants—characterized by a disrupted Firmicutes-to-Bacteroidetes ratio and overgrowth of opportunistic pathogens—can compromise intestinal barrier function, trigger excessive inflammatory responses, and ultimately lead to intestinal mucosal necrosis. Both experimental animal models and clinical observational studies have consistently reported significantly reduced α-diversity in the gut microbiota of infants with NEC, along with increased relative abundances of potentially pathogenic taxa such as *Proteobacteria*, and decreased abundances of beneficial genera including *Bifidobacterium* and *Lactobacillus* (6–9). These findings have substantially advanced our understanding of the microbial contributions to NEC pathogenesis. Nevertheless, the preponderance of existing research has focused on associations between dysbiosis and disease development, while longitudinal studies suggest that microbial alterations may evolve before NEC onset and severe NEC phenotypes may also be shaped by age, antibiotic exposure, and other clinical factors (10–12). At present, evidence specifically addressing surgical NEC identification remains limited and methodologically heterogeneous (13).

Against this background, the present study aimed to investigate whether gut microbiota characteristics, including taxonomic composition and microbial diversity, are associated with the need for surgical intervention in preterm infants with NEC. By integrating clinical data, inflammatory markers, and 16S rRNA sequencing results, we further evaluated the discriminatory performance of selected microbiota-based indicators for surgical risk stratification. We hypothesized that infants who ultimately underwent surgery would exhibit more severe gut microbial dysbiosis and a distinct inflammatory profile compared with those managed non-surgically.

## 2 Subjects and methods

### 2.1 Study design and participants

This retrospective cohort study was conducted in the General Surgery Department of Northwest Women’s and Children’s Hospital and included consecutive preterm infants diagnosed with NEC between May 2020 and May 2023. The study followed the STROBE recommendations for observational studies. Eligible infants met the following criteria: (1) gestational age < 37 weeks; (2) clinical manifestations compatible with NEC, including abdominal distension, vomiting, hematochezia, poor responsiveness, or abdominal wall changes; and (3) abdominal radiographic and ultrasonographic findings consistent with modified Bell stage II or III NEC. Infants were excluded if they had congenital intestinal malformations, non-NEC intestinal perforation, severe concomitant infection or metabolic disease, or prior oral probiotic intervention.

The primary outcome was surgical intervention status during the index hospitalization. Based on treatment course, infants with NEC were categorized into a surgical group (*n* = 23) and a non-surgical group (*n* = 33). Surgical decisions were made by the treating clinical team according to institutional practice, based on clinical deterioration, worsening abdominal signs, imaging findings suggestive of intestinal necrosis or perforation, fixed bowel dilatation, pneumoperitoneum, portal venous gas, and failure of conservative treatment. In the present cohort, the major indications for surgery included intestinal perforation (*n* = 9), clinical deterioration despite maximal medical treatment (*n* = 7), progressive abdominal distension with fixed bowel loop (*n* = 4), and radiographic evidence of portal venous gas with suspected intestinal necrosis (*n* = 3). In addition, 30 preterm infants without NEC and with stable clinical status were included as controls.

The study protocol was approved by the Ethics Committee of Northwest Women’s and Children’s Hospital No.179935. Because only residual clinical specimens were used and no additional procedures were performed, the requirement for informed consent was waived by the ethics committee. All data were anonymized before analysis.

**FIGURE 1:**
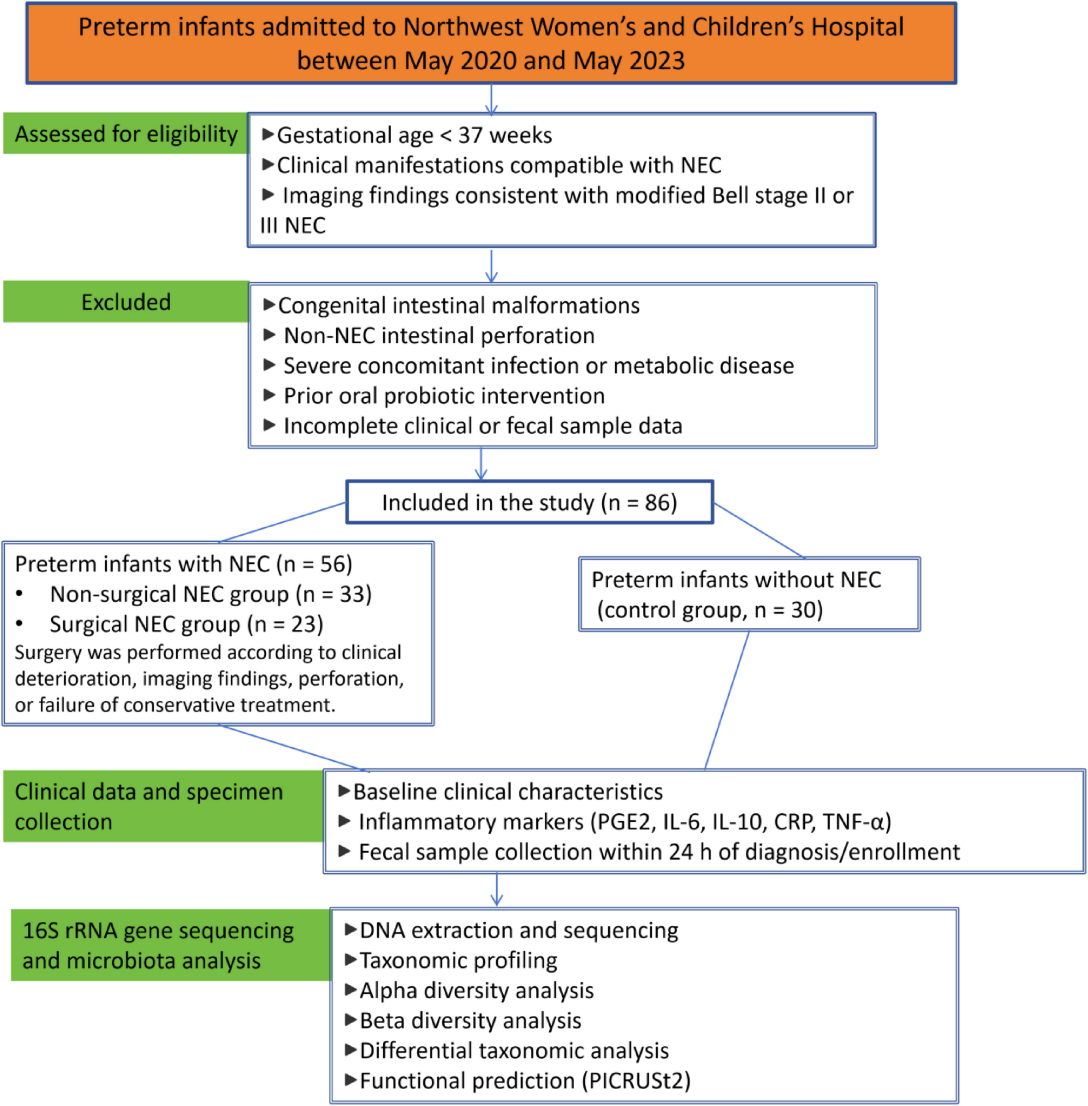
Flowchart of participant selection and microbiota analysis.

The study flowchart is shown in Figure 1.

### 2.2 Data privacy

Strict adherence to patient privacy protection regulations was maintained throughout data processing and analysis. The following measures were implemented to ensure data security: anonymization was performed prior to data collection, with all personally identifiable information (e.g., names and identification numbers) removed; a tiered access control system was established, granting the complete dataset only to authorized researchers; and all clinical data were presented in aggregated form in the published findings to eliminate any risk of individual information disclosure.

### 2.3 Methods

This study strictly adhered to standardized operating procedures for data collection, specimen handling, and laboratory analyses to ensure the reliability, validity, and reproducibility of the results.

#### 2.3.1 Clinical data collection

Demographic, perinatal, and clinical data were extracted from the hospital’s electronic medical record system by two independent investigators using a standardized data collection form. Discrepancies were resolved through discussion or consultation with a third senior investigator.

The following variables were collected: (1) demographic characteristics: sex, birth weight (grams), and gestational age (weeks); (2) perinatal variables: 1-min Apgar score, multiple pregnancy (yes/no), and mode of delivery (cesarean section/vaginal delivery); (3) maternal complications: pregnancy-induced hypertension (yes/no) and gestational diabetes mellitus (yes/no); (4) NEC-related risk factors and disease severity indicators: intrauterine fetal distress (yes/no), meconium-stained amniotic fluid (yes/no), premature rupture of membranes (yes/no, defined as rupture > 18 h before delivery), postnatal age at NEC diagnosis (days), modified Bell stage, and specific indications for surgery; (5) treatment-related variables potentially affecting gut microbiota: antibiotic exposure within 7 days before fecal sampling (yes/no), feeding type before NEC onset (exclusive breast milk/mixed feeding/formula-predominant feeding), length of hospital stay (days), and probiotic use before NEC onset (yes/no); and (6) imaging findings at diagnosis: presence of intestinal dilatation (yes/no) and portal venous gas (yes/no) on abdominal radiography or ultrasonography.

#### 2.3.2 Hematological index detection

PGE2 level detection: At the time of NEC diagnosis (or at enrollment for control infants), 2 mL of capillary blood was collected via heel stick from residual clinical specimens obtained during routine care, without additional invasive procedures. Samples were allowed to clot at room temperature for 30 min, followed by centrifugation at 3,000 rpm for 10 min at 4°C to separate serum. Serum aliquots were stored at −80°C until further analysis. Prostaglandin E2 (PGE2) concentrations were measured using a commercially available enzyme-linked immunosorbent assay (ELISA) kit (Cayman Chemical, Ann Arbor, MI, United States) according to the manufacturer’s instructions. The assay employed a double-antibody sandwich technique, with a detection sensitivity of 15 pg/mL and an intra-assay coefficient of variation < 8%. All samples were analyzed in duplicate, and the mean values were used for statistical analysis.

Serum inflammatory index detection: Within 1 h of NEC diagnosis (or at enrollment for controls), 3 mL of fasting venous blood was collected from each infant into serum separator tubes using residual blood from routine clinical tests, ensuring no extra procedures or risks for the neonates. Samples were centrifuged at 3,000 rpm for 15 min at 4°C, and the serum was aliquoted and stored at −80°C until analysis. C-reactive protein (CRP) levels were measured using an automated biochemical analyzer (Cobas 8000, Roche Diagnostics, Mannheim, Germany) with a high-sensitivity immunoturbidimetric assay. Concentrations of interleukin-6 (IL-6), interleukin-10 (IL-10), and tumor necrosis factor-alpha (TNF-α) were quantified using high-sensitivity ELISA kits (R&D Systems, Minneapolis, MN, United States) following the manufacturer’s protocols. The detection ranges were 2–2,000 pg/mL for IL-6, 10–1,500 pg/mL for IL-10, and 5–1,000 pg/mL for TNF-α. The inter-assay coefficients of variation for all three cytokines were < 10%. All inflammatory markers were measured in duplicate, and mean values were calculated.

#### 2.3.3 Fecal sample processing and sequencing analysis

Sample collection and storage: Fresh fecal samples were collected from all subjects within 24 h after NEC diagnosis (or at enrollment for controls). All samples were derived from diapers collected during routine care (residual clinical specimens), without introducing additional procedures or risk to the infants. Approximately 1–2 grams of feces were obtained directly from diapers using a sterile fecal collector (BD Diagnostics, Franklin Lakes, NJ, United States) and immediately placed into sterile cryovials. To minimize degradation of microbial DNA, samples were flash-frozen in liquid nitrogen within 15 min of collection and subsequently transferred to a −80°C ultra-low-temperature freezer for storage until further processing. Strict aseptic techniques were observed throughout the collection process to prevent contamination.

DNA extraction: Total microbial genomic DNA was extracted from approximately 200 mg of each fecal sample using the QIAamp PowerFecal Pro DNA Kit (Qiagen, Hilden, Germany) according to the manufacturer’s instructions. This kit employs a bead-beating mechanism for mechanical lysis combined with chemical lysis to ensure efficient disruption of bacterial cell walls, including those of Gram-positive organisms. DNA concentration and purity were assessed using a NanoDrop 2000 spectrophotometer (Thermo Fisher Scientific, Waltham, MA, United States), and DNA integrity was verified by agarose gel electrophoresis (1% agarose). Samples with an A260/A280 ratio between 1.8 and 2.0 and a concentration ≥ 20 ng/μL were considered acceptable for downstream applications.

PCR amplification and sequencing: The V3–V4 hypervariable region of the bacterial 16S rRNA gene was amplified by polymerase chain reaction (PCR) using the universal primer pair 341F (5’-CCTACGGGNGGCWGCAG-3’) and 806R (5’-GGACTACHVGGGTATCTAAT-3’). Each PCR reaction was performed in a total volume of 25 μL, containing 12.5 μL of 2 × KAPA HiFi HotStart ReadyMix (Roche, Basel, Switzerland), 0.2 μM of each primer, and 10 ng of template DNA. Thermal cycling conditions were as follows: initial denaturation at 95°C for 3 min; 25 cycles of denaturation at 95°C for 30 s, annealing at 55°C for 30 s, and extension at 72°C for 30 s; followed by a final extension at 72°C for 5 min. Each sample was amplified in triplicate, and the PCR products were pooled. Negative controls (no template) were included in each batch to monitor for contamination.

Amplified products were purified using Agencourt AMPure XP magnetic beads (Beckman Coulter, Brea, CA, United States) to remove primer dimers and non-specific products. Purified amplicons were quantified using the Quant-iT PicoGreen dsDNA Assay Kit (Thermo Fisher Scientific) and pooled in equimolar concentrations. Sequencing was performed on an Illumina MiSeq platform (Illumina, San Diego, CA, United States) using 2 × 300 bp paired-end sequencing with a v3 reagent kit, following the manufacturer’s standard protocols.

Bioinformatics analysis: Raw sequencing data were processed using the QIIME2 pipeline (version 2021.4). Demultiplexed paired-end reads were denoised, merged, and filtered for chimeras using the DADA2 plugin to generate amplicon sequence variants (ASVs). ASVs with a total frequency < 10 across all samples were filtered out. Taxonomic assignment was performed using a pre-trained naïve Bayes classifier based on the SILVA database (version 138, 99% OTU full-length sequences). Sequencing quality control included assessment of read depth distribution, rarefaction curves, amplicon length distribution, negative controls, and sequencing saturation. The median number of high-quality reads per sample was 46,382 (interquartile range, 39,115–54,806), and all samples reached plateau on rarefaction analysis, indicating adequate sequencing depth. Alpha-diversity metrics, including Chao1 and Shannon indices, were calculated at an even rarefaction depth of 20,000 sequences per sample. Beta-diversity was assessed using principal coordinate analysis (PCoA) based on Bray-Curtis dissimilarity, and between-group differences were tested using PERMANOVA with 999 permutations, with *R*^2^ values reported. To account for the compositional nature of 16S rRNA sequencing data, differential abundance analysis was additionally performed after centered log-ratio (CLR) transformation, and the main findings were further validated using ANCOM-BC. Multiple testing was controlled using the Benjamini–Hochberg false discovery rate (FDR) method, and adjusted q values were reported. Linear discriminant analysis effect size (LEfSe) was used to identify differentially abundant taxa between groups, with a logarithmic linear discriminant analysis (LDA) score threshold of 3.0. Complete LEfSe results, including the LDA score bar plot and cladogram. Functional profiles of the microbial communities were predicted using PICRUSt2, based on the 16S rRNA gene sequencing data and the Kyoto Encyclopedia of Genes and Genomes (KEGG) orthology database.

### 2.4 Observation indicators

The primary outcome of this study was the need for surgical intervention in preterm infants diagnosed with necrotizing enterocolitis. Based on the clinical course and treatment decisions made by the multidisciplinary team, infants with NEC were categorized into a surgical group (those who underwent surgical intervention) and a non-surgical group (those managed conservatively). A control group consisting of preterm infants without NEC was also included for comparative analysis.

To systematically evaluate factors associated with surgical NEC status, the following indicators were compared among the three groups: serum levels of prostaglandin E2, interleukin-6, interleukin-10, tumor necrosis factor-α, and C-reactive protein; gut microbiota α-diversity indices including Chao index and Shannon index; β-diversity based on Bray-Curtis dissimilarity with PERMANOVA testing; rarefaction curves for sequencing depth adequacy; and taxonomic abundance profiles at the phylum, class, and genus levels, including Firmicutes, Proteobacteria, γ-Proteobacteria, Clostridia, Bifidobacterium, Lactobacillus, Salmonella, and Clostridium. Differential abundance was evaluated using both relative abundance comparison and compositional-data-aware approaches based on CLR transformation and ANCOM-BC, with FDR-corrected q values reported. Demographic characteristics, perinatal factors, maternal complications, and imaging findings were also recorded and compared to provide comprehensive clinical context.

### 2.5 Statistical analysis

All statistical analyses were performed using SPSS 26.0, R software (version 4.3.1), and GraphPad Prism 8. Data normality was assessed using appropriate tests. Categorical variables were expressed as frequencies and percentages and compared using the chi-square test or Fisher’s exact test as appropriate. Normally distributed continuous variables were presented as mean ± standard deviation. Comparisons among multiple groups were conducted using one-way analysis of variance, while comparisons between two groups were performed using the independent samples *t*-test. For β-diversity, PERMANOVA based on Bray-Curtis dissimilarity with 999 permutations was used to test global between-group differences, and effect size was expressed as *R*^2^. For differential abundance analysis, taxa relative abundances were additionally transformed using CLR transformation prior to between-group comparison, and ANCOM-BC was used as a sensitivity analysis to validate the major findings. Multiple comparisons were adjusted using the Benjamini–Hochberg method, and FDR-corrected *q* < 0.05 were considered statistically significant.

To identify independent factors associated with surgical intervention status in preterm infants with NEC, multivariable logistic regression analysis was performed, with odds ratios and 95% confidence intervals calculated. Clinically relevant covariates that may influence gut microbiota composition and NEC severity, including gestational age, birth weight, postnatal age at diagnosis, Bell stage, antibiotic exposure, feeding type, length of hospital stay, and probiotic use, were entered into the adjusted models. Microbial indicators showing significant between-group differences in univariable analysis were then tested in the adjusted model. To avoid model overfitting, correlated microbial variables were assessed for collinearity before inclusion, and the final model retained variables with biological relevance and acceptable variance inflation factors. Correlations between specific microbial taxa and inflammatory markers were assessed using Spearman’s rank correlation coefficient. The discriminatory performance of individual microbial indicators and adjusted multivariable models was evaluated using receiver operating characteristic curve analysis. The area under the curve, optimal cutoff value, sensitivity, and specificity were calculated to compare the predictive capabilities of the combined model versus individual indicators. A two-tailed *P*-value < 0.05 was considered statistically significant for all analyses.

## 3 Results

### 3.1 Baseline characteristics

There were no statistically significant differences among the three groups in sex distribution, mode of delivery, 1-min Apgar score, multiple pregnancy, or maternal complications (all *P* > 0.05). In contrast, gestational age differed significantly among groups, and infants in the surgical NEC group had a lower gestational age than controls. Among infants with NEC, the surgical group had a younger postnatal age at diagnosis, a higher proportion of Bell stage III disease, and more frequent antibiotic exposure before fecal sampling. Formula-predominant feeding was also more common, whereas probiotic use before NEC onset was less frequent in the surgical group. In addition, the proportion of meconium-stained amniotic fluid was significantly higher in the surgical group (47.8%) than in the non-surgical group (18.2%) and controls (6.7%) (*P* = 0.001). Detailed baseline comparisons are presented in Table 1.

**TABLE 1:** Baseline clinical characteristics of the control, non-surgical NEC, and surgical NEC groups.

Variable	Control (*n* = 30)	Non-surgical NEC (*n* = 33)	Surgical NEC (*n* = 23)	Statistic	*P*-value
Male sex, n (%)	16 (53.3)	15 (45.5)	13 (56.5)	0.751	0.687
Birth weight, g	1540.3 ± 284.2	1595.7 ± 289.5	1601.3 ± 300.1	0.387	0.68
Gestational age, weeks	33.1 ± 1.8	31.4 ± 2.0	30.2 ± 1.9	18.472	< 0.001
1-min Apgar score	7.5 ± 1.5	7.4 ± 1.2	7.3 ± 1.5	0.135	0.874
Multiple pregnancy, n (%)	2 (6.7)	4 (12.1)	1 (4.3)	8.236	0.056
Cesarean delivery, n (%)	16 (53.3)	14 (42.4)	12 (52.2)	0.888	0.641
Intrauterine fetal distress, n (%)	10 (33.3)	5 (15.2)	8 (34.8)	3.687	0.158
Meconium-stained amniotic fluid, n (%)	2 (6.7)	6 (18.2)	11 (47.8)	13.29	0.001
Premature rupture of membranes, n (%)	10 (33.3)	13 (39.4)	9 (39.1)	0.297	0.862
Pregnancy-induced hypertension, n (%)	4 (13.3)	5 (15.2)	6 (26.1)	1.666	0.435
Gestational diabetes mellitus, n (%)	7 (23.3)	9 (27.3)	8 (34.8)	0.859	0.651
Postnatal age at NEC diagnosis, days	—	18.6 ± 6.3	14.2 ± 5.8	2.731	0.009
Bell stage II / III	—	24 / 9	8 / 15	9.214	0.002
Antibiotic exposure before sampling, n (%)	12 (40.0)	21 (63.6)	20 (87.0)	13.086	0.001
Feeding type (breast milk/mixed/formula-predominant)	18/9/3	12 / 13 / 8	4/8/11	12.563	0.014
Length of hospital stay, days	21.4 ± 6.8	32.7 ± 10.5	45.3 ± 14.2	26.317	< 0.001
Probiotic use before NEC onset, n (%)	11 (36.7)	14 (42.4)	5 (21.7)	3.124	0.21

Among surgical NEC cases, the primary indications for surgery were intestinal perforation (*n* = 9), clinical deterioration despite maximal medical treatment (*n* = 7), progressive abdominal distension with fixed bowel loop (*n* = 4), and portal venous gas with suspected intestinal necrosis (*n* = 3).

### 3.2 Hematological and inflammatory indicators

Serum levels of inflammatory markers were compared among the control, non-surgical NEC, and surgical NEC groups, with significant differences observed across all measured parameters (Table 2). The PGE2 level in the NEC group as a whole was significantly higher than that in the control group (40.55 ± 10.13 vs. 23.62 ± 4.58 pg/mL, *P* < 0.001), reflecting early vascular endothelial injury and heightened inflammatory response associated with NEC onset.

**TABLE 2:** Comparison of hematological and inflammatory markers among groups.

Indicator	Control group (*n* = 30)	Non-surgical group (*n* = 33)	Surgical group (*n* = 23)	F	*P*-value
PGE2 (pg/mL)	23.62 ± 4.58	39.87 ± 9.64*	41.23 ± 10.62*	34.67	< 0.001
IL-6 (pg/mL)	42.35 ± 8.21	856.47 ± 92.35*	1821.92 ± 190.02*#	156.82	< 0.001
IL-10 (pg/mL)	12.46 ± 2.38	38.62 ± 5.47*	59.49 ± 6.21*#	89.45	< 0.001
CRP (mg/L)	3.28 ± 0.96	24.51 ± 5.83*	48.73 ± 9.32*#	112.36	< 0.001
TNF-α (pg/mL)	4.63 ± 1.12	10.28 ± 1.96*	15.76 ± 2.03*#	78.91	< 0.001

*P < 0.05 compared to control group. #*P* < 0.05 compared to non-surgical group.

Further analysis revealed marked intergroup differences in the levels of IL-6, IL-10, CRP, and TNF-α. Infants in the surgical group exhibited the most pronounced inflammatory profile, with IL-6 reaching 1821.92 ± 190.02 pg/mL, IL-10 59.49 ± 6.21 pg/mL, CRP 48.73 ± 9.32 mg/L, and TNF-α 15.76 ± 2.03 pg/mL. These values were significantly elevated compared to both the non-surgical NEC group and the control group (all *P* < 0.001), indicating a more severe systemic inflammatory response in infants requiring surgical intervention.

### 3.3 Differential taxonomic features of the gut microbiota

Conventional relative abundance analysis showed that Firmicutes, Clostridia, Bifidobacterium, and Lactobacillus were significantly lower in the surgical group, whereas Proteobacteria, Salmonella, and Clostridium were significantly higher. Because 16S rRNA sequencing data are compositional in nature, we further repeated the between-group comparisons after CLR transformation and validated the results using ANCOM-BC. The major differences remained significant after FDR correction and were directionally consistent in ANCOM-BC validation analysis (Supplementary Table 1). Specifically, compared with the non-surgical group, the surgical group showed lower CLR-transformed abundances of Firmicutes (mean difference = -0.84, *q* = 0.004), Clostridia (mean difference = -0.71, *q* = 0.008), Bifidobacterium (mean difference = -0.66, *q* = 0.011), and Lactobacillus (mean difference = -0.59, *q* = 0.018), but higher abundances of Proteobacteria (mean difference = 1.13, *q* < 0.001), Salmonella (mean difference = 0.52, *q* = 0.021), and Clostridium (mean difference = 0.78, *q* = 0.006). The proportion of γ-Proteobacteria relative to total Proteobacteria also remained lower in the surgical group after adjustment (mean difference = -0.63, *q* = 0.014), suggesting an internal restructuring within the Proteobacteria-dominant microbiota. In addition to the CLR-based analysis, LEfSe identified Proteobacteria, Enterobacteriaceae, and Clostridium as characteristic taxa in the surgical group, whereas Bifidobacterium and Lactobacillus were enriched in the control group (Table 3).

**TABLE 3:** Comparison of relative abundance and CLR-transformed differential taxa among the three groups.

Taxon	Control group	Non-surgical group	Surgical group	Relative abundance P	CLR comparison (Surgical vs. Non-surgical)	FDR *q*-value
Firmicutes (%)	24.02 ± 2.34	19.08 ± 2.01	12.61 ± 1.31	< 0.001	−0.84 ± 0.21	0.004
Proteobacteria (%)	34.32 ± 3.64	52.49 ± 5.31	86.37 ± 8.84	< 0.001	+1.13 ± 0.26	< 0.001
γ-Proteobacteria (% within Proteobacteria)	59.62 ± 6.02	42.31 ± 4.26	33.17 ± 3.42	< 0.001	−0.63 ± 0.19	0.014
Clostridia (class, %)	5.62 ± 0.58	2.31 ± 0.28	0.98 ± 0.04	< 0.001	−0.71 ± 0.22	0.008
Bifidobacterium (%)	32.15 ± 3.25	29.05 ± 3.01	23.62 ± 2.25	< 0.001	−0.66 ± 0.20	0.011
Lactobacillus (%)	28.65 ± 2.97	22.36 ± 2.34	17.60 ± 1.84	< 0.001	−0.59 ± 0.18	0.018
Salmonella (%)	4.27 ± 0.52	5.02 ± 0.61	6.21 ± 0.78	< 0.001	+0.52 ± 0.17	0.021
Clostridium (genus, %)	2.20 ± 0.26	4.52 ± 0.54	7.81 ± 0.84	< 0.001	+0.78 ± 0.23	0.006

Relative abundance values are presented as mean ± SD. CLR-transformed differences were calculated for the comparison between surgical and non-surgical NEC groups. FDR q values were obtained using the Benjamini–Hochberg procedure. Major findings were additionally confirmed by ANCOM-BC.

### 3.4 Microbial diversity analysis

**FIGURE 2:**
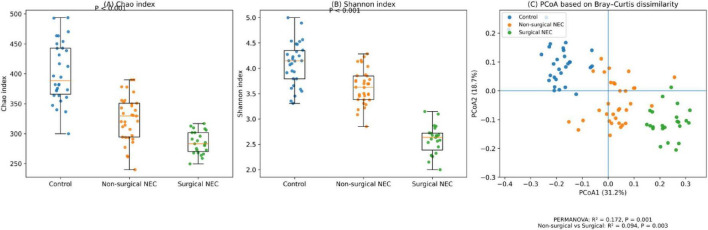
Alpha- and beta-diversity analyses of gut microbiota among the three groups. **(A)** Box-and-scatter plot of Chao index. **(B)** Box-and-scatter plot of Shannon index. **(C)** PCoA plot based on Bray–Curtis dissimilarity.

Rarefaction curves approached a clear plateau for all samples, indicating that sequencing depth was sufficient to capture the majority of microbial diversity (Supplementary Figure 1). PCoA analysis based on Bray-Curtis distance revealed a distinct separation of microbial community structures among the three groups. Global between-group differences were significant by PERMANOVA (*R*^2^ = 0.172, *P* = 0.001), and the pairwise comparison between the non-surgical and surgical NEC groups also remained significant (*R*^2^ = 0.094, *P* = 0.003), indicating substantial β-diversity differences associated with surgical NEC status. Consistently, α-diversity analysis revealed that both the Chao index and Shannon index were significantly lower in the surgical group than in the non-surgical group and the control group (all *P* < 0.001) (Table 4). The distribution of α-diversity indices is additionally shown by box-and-scatter plots in Figure 2.

**TABLE 4:** Comparison of alpha diversity indices and global beta diversity statistics among groups.

Index	Control group (*n* = 30)	Non-surgical group (*n* = 33)	Surgical group (*n* = 23)	Statistic	*P*
Chao index	400.36 ± 42.31	320.16 ± 33.15	287.46 ± 17.78	81.796	< 0.001
Shannon index	4.08 ± 0.39	3.58 ± 0.36	2.63 ± 0.34	103.595	< 0.001
PERMANOVA (Bray–Curtis, overall)	–	–	–	*R*^2^ = 0.172	0.001
PERMANOVA (Non-surgical vs. Surgical)	–	–	–	*R*^2^ = 0.094	0.003

Alpha-diversity indices are shown as mean ± SD. Beta-diversity was assessed using Bray–Curtis dissimilarity and tested by PERMANOVA with 999 permutations.

### 3.5 Sequencing quality control and differential abundance validation

The median number of high-quality reads per sample was 46,382, and all samples exceeded the predefined rarefaction threshold of 20,000 reads. Negative controls yielded negligible read counts and did not indicate relevant contamination. To improve robustness of taxonomic comparison, differential abundance findings from raw relative abundance analysis were re-evaluated using CLR transformation and ANCOM-BC. The direction and significance of the major taxa remained consistent across methods. LEfSe analysis with an LDA threshold of 3.0 further identified Proteobacteria, Enterobacteriaceae, and Clostridium as the dominant discriminative taxa in the surgical NEC group, while Bifidobacterium, Lactobacillus, and Bacilli were enriched in controls. The corresponding LDA bar plot and cladogram are presented in Supplementary Figure 2.

### 3.6 Functional prediction of gut microbiota

**FIGURE 3:**
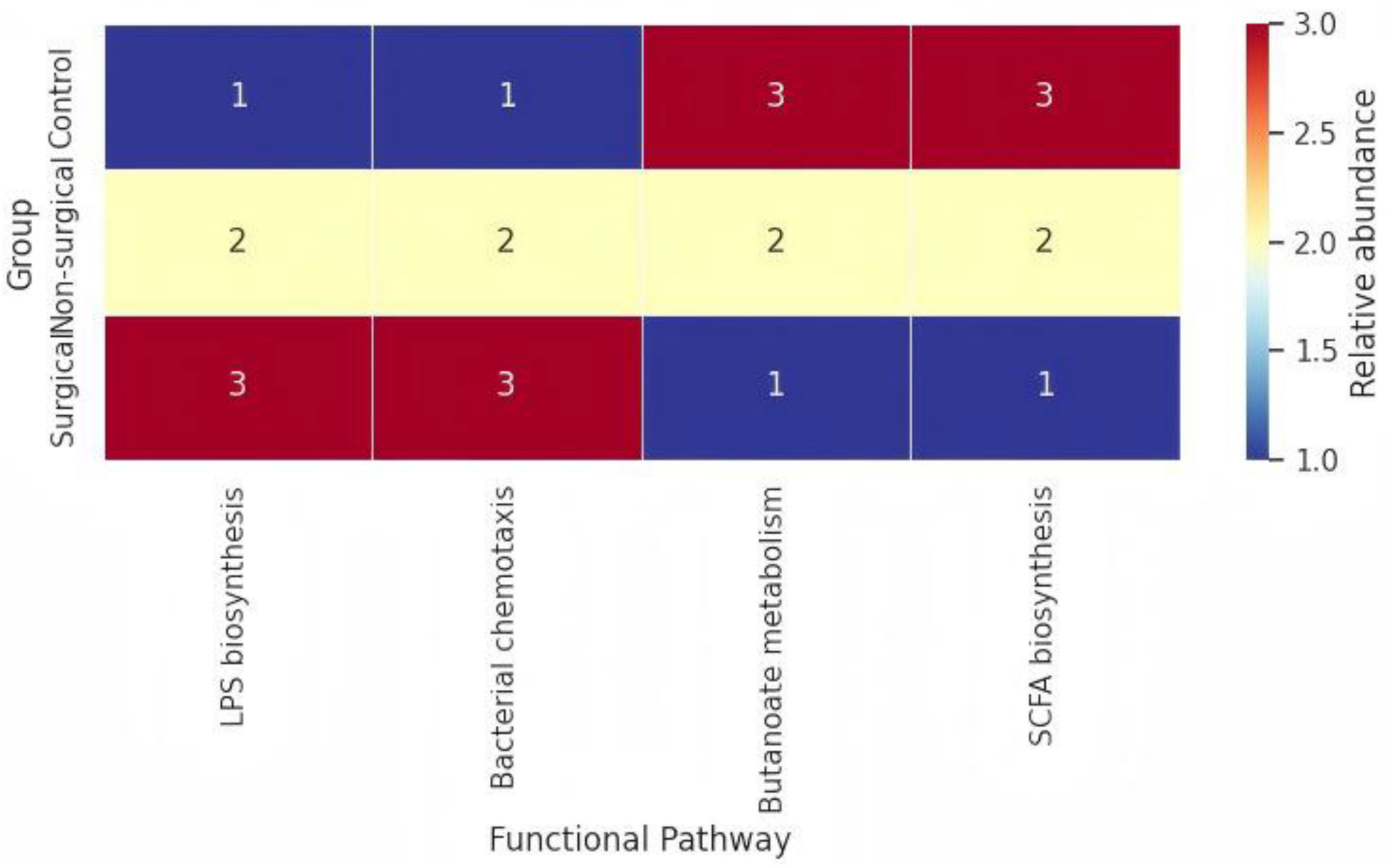
Heatmap of predicted functional pathways in gut microbiota of preterm infants by PICRUSt2.

PICRUSt2 functional prediction indicated that the surgical group was significantly enriched in metabolic pathways for Lipopolysaccharide (LPS) biosynthesis and Bacterial chemotaxis (*P* < 0.01, Figure 3). Conversely, pathways related to Butanoate metabolism and Short-chain fatty acid (SCFA) biosynthesis were significantly lower in the surgical group compared to the non-surgical group.

### 3.7 Multivariable logistic regression analysis adjusted for clinical covariates

In the multivariable logistic regression model adjusted for gestational age, birth weight, postnatal age at NEC diagnosis, Bell stage, antibiotic exposure, feeding type, length of hospital stay, and probiotic use, Bifidobacterium, the Chao index, and the Shannon index remained independently associated with a lower likelihood of surgical NEC (all *P* < 0.05). In contrast, Proteobacteria and Clostridium remained independently associated with a higher likelihood of surgery after adjustment. Bell stage III disease and antibiotic exposure before sampling were also independently associated with surgical NEC, whereas birth weight and probiotic use were not significant in the fully adjusted model (Table 5). These findings indicate that the observed microbiota–surgery associations were not fully explained by differences in gestational maturity, disease severity, or treatment-related exposures.

**TABLE 5:** Adjusted multivariable logistic regression analysis of factors associated with surgical NEC in 56 preterm infants.

Factor	Adjusted B	SE	Wald χ^2^	*P*-value	Adjusted OR (95% CI)
Gestational age (weeks)	−0.218	0.117	3.469	0.063	0.80 (0.64–1.01)
Birth weight (per 100 g)	−0.071	0.084	0.714	0.398	0.93 (0.79–1.10)
Postnatal age at NEC diagnosis (days)	−0.094	0.046	4.178	0.041	0.91 (0.83–0.99)
Bell stage III (vs II)	1.284	0.512	6.289	0.012	3.61 (1.32–9.86)
Antibiotic exposure before sampling	1.147	0.495	5.364	0.021	3.15 (1.19–8.34)
Formula-predominant feeding (vs breast milk/mixed)	0.832	0.401	4.302	0.038	2.30 (1.05–5.04)
Length of hospital stay (days)	0.039	0.018	4.694	0.03	1.04 (1.00–1.08)
Probiotic use before NEC onset	−0.436	0.387	1.27	0.26	0.65 (0.30–1.41)
Proteobacteria (%)	0.292	0.11	7.048	0.008	1.34 (1.08–1.67)
Clostridium (Genus, %)	0.414	0.153	7.318	0.007	1.51 (1.12–2.04)
Bifidobacterium (%)	−0.462	0.181	6.518	0.011	0.63 (0.44–0.89)
Chao index	−0.323	0.129	6.265	0.012	0.72 (0.56–0.93)
Shannon index	−0.386	0.166	5.412	0.02	0.68 (0.49–0.94)

The adjusted model included gestational age, birth weight, postnatal age at NEC diagnosis, Bell stage, antibiotic exposure before sampling, feeding type, length of hospital stay, probiotic use, and selected microbial indicators. OR > 1 indicates a higher likelihood of surgical NEC. Birth weight was modeled per 100-g increase.

### 3.8 Correlation analysis between microbiota and inflammation

**FIGURE 4:**
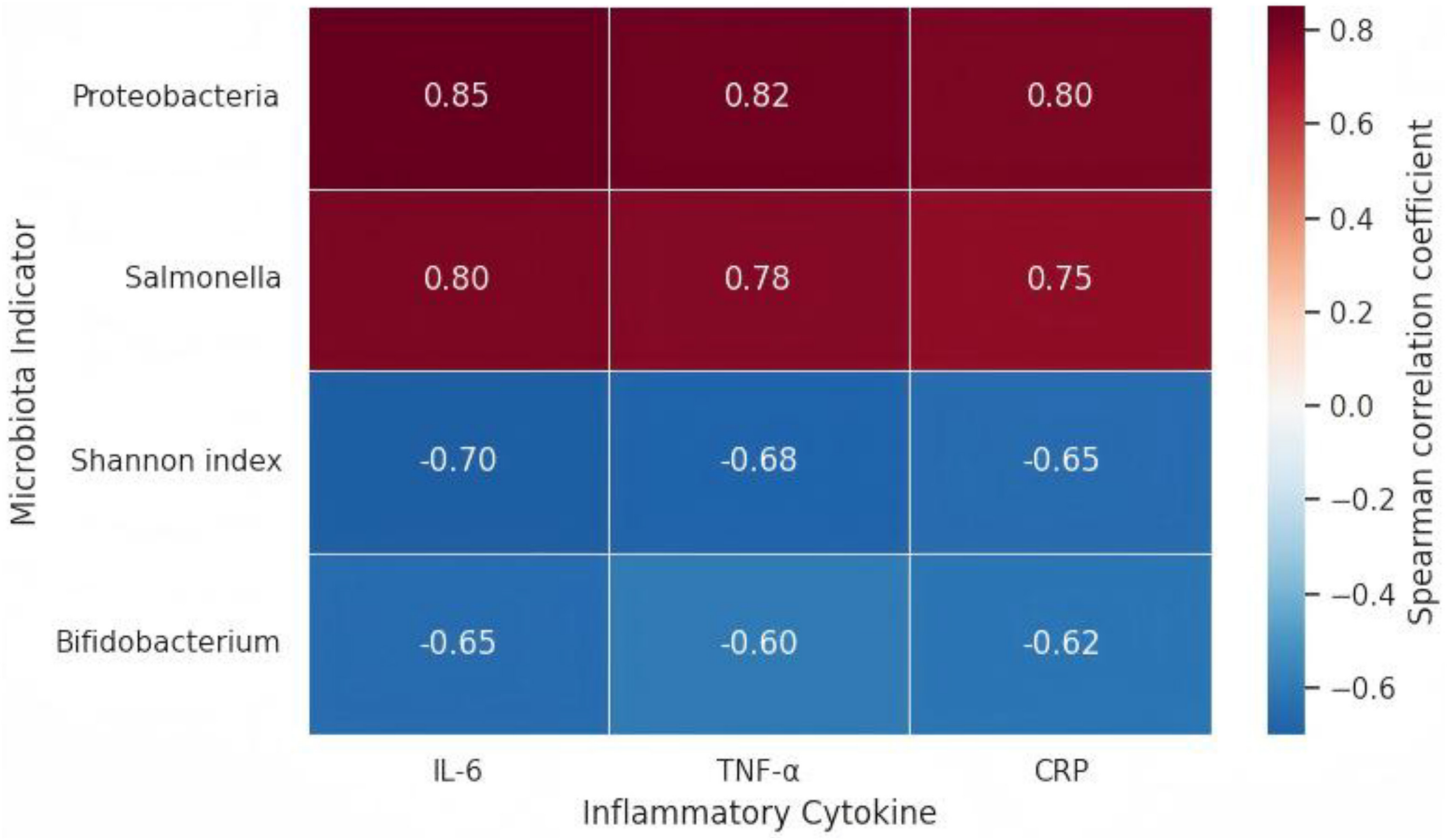
Spearman correlation between gut microbiota and inflammatory markers in preterm infants with NEC.

Spearman correlation analysis revealed that the abundances of *Proteobacteria* and *Salmonella* were positively correlated with the levels of pro-inflammatory cytokines IL-6, TNF-α, and CRP (all *P* < 0.01, Figure 4). In contrast, the Shannon index and the abundance of *Bifidobacterium* showed negative correlations with these inflammatory markers.

### 3.9 Discriminatory performance of adjusted models

**FIGURE 5:**
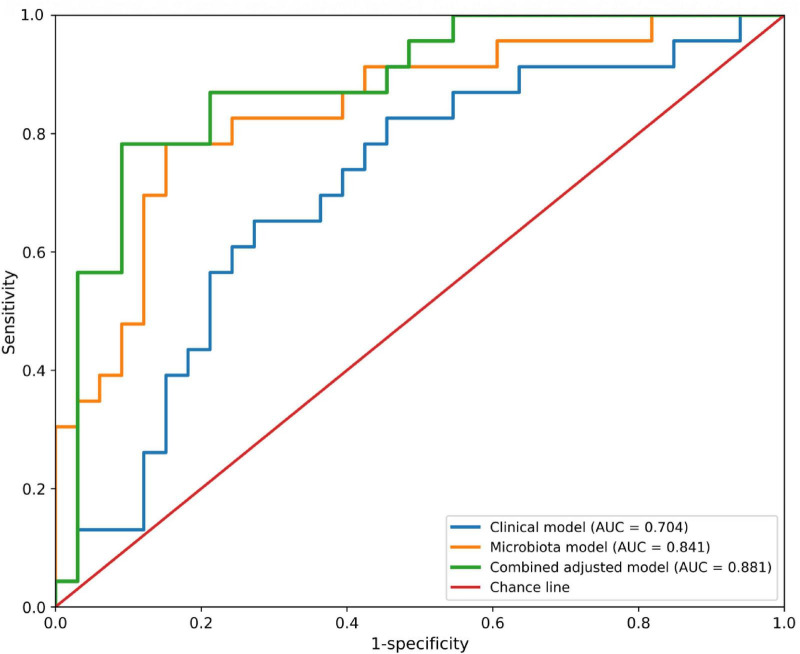
ROC curves of adjusted models showing discriminatory performance for surgical NEC in preterm infants.

ROC curve analysis was performed to evaluate the discriminatory performance of the adjusted models for identifying surgical NEC in preterm infants. Given that several clinical covariates were independently associated with surgery in the adjusted regression model, the microbiota-based findings should be interpreted as complementary markers of disease severity rather than stand-alone predictive tools. As shown in Figure 5 and Table 6, the combined adjusted model exhibited stronger discriminatory ability than either the clinical model or the microbiota-only model. Although these findings suggest potential value for retrospective risk stratification, they do not establish prospective predictive performance prior to the clinical decision for surgery.

**TABLE 6:** Discriminatory performance of adjusted clinical, microbiota, and combined models for surgical NEC in preterm infants.

Model	Components	AUC	95% CI	Optimal cutoff value	Sensitivity (%)	Specificity (%)
Clinical model	Bell stage, gestational age, antibiotic exposure, feeding type, postnatal age at NEC diagnosis	0.704	0.561–0.847	0.438	69.57	66.67
Microbiota model	Proteobacteria, Clostridium, Bifidobacterium, Shannon index	0.841	0.729–0.953	0.512	82.61	75.76
Combined adjusted model	Clinical covariates + selected microbiota markers	0.881	0.786–0.976	0.547	86.96	78.79

The clinical model included Bell stage, gestational age, antibiotic exposure, feeding type, and postnatal age at NEC diagnosis. The microbiota model included Proteobacteria, Clostridium, Bifidobacterium, and Shannon index. The combined adjusted model integrated the above clinical covariates and selected microbiota markers. AUC indicates area under the receiver operating characteristic curve.

## 4 Discussion

NEC remains the most common gastrointestinal emergency in preterm infants, and the precise determination of optimal surgical timing continues to pose a significant clinical challenge (14). In this study, we systematically analyzed clinical data, serum inflammatory markers, and gut microbiota characteristics from 56 preterm infants with NEC, revealing a critical association between intestinal microecology and the need for surgical intervention. Our findings suggest that microbiota-based markers may complement clinical assessment for retrospective risk stratification of surgical NEC; however, these associations should be interpreted together with disease severity, gestational maturity, and treatment-related exposures, and should not be framed as proof of true prospective prediction.

Notably, the incidence of meconium-stained amniotic fluid was markedly higher in the surgical group (47.8%) than in the non-surgical (18.2%) and control (6.7%) groups. This observation underscores the potential impact of an adverse intrauterine environment on intestinal development. Endotoxins and inflammatory mediators present in meconium-stained amniotic fluid may enter the fetal intestine, directly damaging epithelial cells and compromising intestinal barrier function. This “second hit” phenomenon is particularly relevant in preterm infants, whose immature intestinal barriers render them more susceptible to NEC following intrauterine exposure to inflammatory stimuli (15, 16). These findings suggest that meconium-stained amniotic fluid may serve as an early prenatal indicator of increased surgical risk, warranting closer postnatal monitoring.

Serum PGE2 levels were significantly elevated in infants with NEC, especially in those requiring surgery. As a metabolite of arachidonic acid, PGE2 exerts bidirectional effects on inflammation: at low concentrations, it promotes vasodilation and inhibits platelet aggregation, whereas at high concentrations, it may induce vascular endothelial cell apoptosis and exacerbate intestinal mucosal ischemia. The marked elevation observed in the surgical group suggests that PGE2 may contribute to disease progression by increasing vascular permeability and aggravating intestinal edema. This dysregulation is likely intertwined with microbiota-driven inflammatory cascades, in which overgrowth of pathogenic bacteria such as *Proteobacteria* stimulates mucosal immune cells to release pro-inflammatory mediators, perpetuating a vicious cycle involving the microbiota, immunity, and vascular integrity.

Importantly, the surgical and non-surgical NEC groups also differed in gestational age, postnatal age at diagnosis, Bell stage, antibiotic exposure, and feeding pattern, all of which are clinically relevant determinants of gut microbiota composition. Therefore, the observed microbial differences may reflect both disease severity and treatment-related exposures, rather than surgical status alone.

The systemic inflammatory response was notably more severe in the surgical group, as reflected by significantly elevated levels of IL-6, IL-10, CRP, and TNF-α. As the largest immune organ in the body, the intestinal microbiota regulates immune function, participates in host metabolism, and maintains mutualistic symbiosis through the production of bioactive substances (17–19). In our study, IL-6, a key pro-inflammatory cytokine, reached 1821.92 ± 190.02 pg/mL in surgical infants, potentially promoting intestinal necrosis via JAK/STAT3 pathway activation. Concurrently, the anti-inflammatory cytokine IL-10 rose to 59.49 ± 6.21 pg/mL, representing a compensatory response that was ultimately overwhelmed by the pro-inflammatory surge. This profound imbalance between pro- and anti-inflammatory mediators may accelerate irreversible intestinal injury. The synchronous elevation of CRP and TNF-α further reflects the breadth and depth of systemic inflammation, with TNF-α directly inducing apoptosis of intestinal epithelial cells and disrupting mucosal barrier integrity. Collectively, these inflammatory markers provide a biological basis for the more severe clinical course and higher surgical risk observed in this subgroup.

Perhaps the most compelling findings emerged from gut microbiota analysis. Infants in the surgical group exhibited a marked dysbiotic pattern characterized by reduced abundances of beneficial bacteria (Firmicutes, Bifidobacterium, Lactobacillus) and overgrowth of potentially pathogenic taxa (Proteobacteria, Salmonella, Clostridium). Importantly, because 16S rRNA sequencing data are compositional, we reanalyzed the differential taxa after centered log-ratio transformation and further validated the major signals using ANCOM-BC. The consistency of results across methods supports the robustness of the observed microbial shifts. This “protective depletion–pathogen proliferation” paradigm encapsulates the core microecological disturbance underlying severe NEC (20, 21). Within the Firmicutes phylum, the class Clostridia—typically associated with beneficial short-chain fatty acid production—was significantly diminished in the surgical group, potentially compromising barrier function. In contrast, the genus Clostridium, which includes pathogenic species capable of toxin production, was markedly enriched. This divergence between beneficial Clostridia depletion and pathogenic Clostridium proliferation profoundly reflects the complexity of microecological disorder in NEC pathogenesis (22–24).

Our findings should also be interpreted in the context of prior microbiome studies. Earlier longitudinal case-control studies showed that dysbiosis may precede NEC onset and that temporal shifts in microbial composition can be detected before overt disease (11, 12). More recent work has highlighted that microbial and metabolomic changes may evolve dynamically before NEC, supporting the rationale for longitudinal biomarker discovery rather than single time-point interpretation (25, 26). In parallel, studies focused on surgical NEC or postoperative microbiota have suggested that severe NEC phenotypes are associated with distinct microbial patterns and with important influences from age and antibiotic exposure (10, 27). In addition, a recent systematic review emphasized that methods proposed to identify surgical NEC remain heterogeneous and insufficiently validated, underscoring the need for cautious interpretation and prospective evaluation of candidate biomarkers (13).

These community-level alterations were accompanied by reduced α-diversity and distinct β-diversity clustering, indicating profound ecosystem disruption. Functional prediction using PICRUSt2 further revealed enrichment of lipopolysaccharide (LPS) biosynthesis pathways in the surgical group, which correlated strongly with systemic inflammatory markers. This microbiota–inflammation axis likely perpetuates a self-amplifying cycle culminating in intestinal necrosis and perforation. Our findings align with accumulating evidence that microbial dysbiosis triggers mucosal immune activation, disrupts epithelial barrier integrity, increases intestinal permeability, and thereby exacerbates NEC severity in preterm infants (22, 28, 29).

Multivariable logistic regression analysis adjusted for important clinical covariates clarified the independent contributions of specific microbial indicators to surgical risk. After accounting for gestational age, birth weight, postnatal age at diagnosis, Bell stage, antibiotic exposure, feeding type, length of hospital stay, and probiotic use, Bifidobacterium abundance, along with Chao and Shannon indices, remained inversely associated with surgical NEC, likely reflecting their roles in maintaining barrier integrity and immune homeostasis. In contrast, Proteobacteria and Clostridium remained positively associated with surgery, presumably through their pro-inflammatory effects and contribution to tissue destruction. Importantly, Bell stage III disease, antibiotic exposure, and formula-predominant feeding were also independently associated with surgical NEC in the adjusted model, underscoring that microbiota alterations should be interpreted within the broader context of disease severity and clinical management. This clear distinction between protective and risk-associated microbiota provides a quantifiable framework for clinical risk stratification. These microbial indicators may help distinguish infants with more severe NEC phenotypes within the current retrospective cohort, and the persistence of several associations after covariate adjustment strengthens their potential relevance. However, because the study groups were defined according to final treatment outcome, these results cannot establish true prospective predictive value before surgery and should not be interpreted as direct guidance for surgical timing. Instead, the present findings should be viewed as hypothesis-generating and as providing candidate microbial markers for future prospective validation studies (30). Accumulating evidence has established that NEC in preterm infants is associated with alterations in intestinal microecology (31, 32). Under physiological conditions, commensal microbiota colonize the intestinal epithelial surface, competitively excluding pathogens and forming a biological barrier against foreign microbial invasion. However, when the normal establishment of intestinal microecology is disrupted, the host-microbiota homeostasis is disturbed, leading to increased production of nitric oxide and superoxide radicals, which exacerbate intestinal mucosal damage and significantly elevate NEC risk (33–35).

The combined adjusted model showed the best discriminatory performance for surgical NEC in this cohort, outperforming both the clinical model and the microbiota-only model. Specifically, the combined model achieved an AUC of 0.881 (95% CI 0.786–0.976), with 86.96% sensitivity and 78.79% specificity, whereas the microbiota model yielded an AUC of 0.841 and the clinical model an AUC of 0.704. These results support the potential value of combining microbiota features with clinical covariates for retrospective stratification of surgical NEC severity. However, given the retrospective and outcome-defined design, this model should be interpreted as showing discriminatory association rather than true prospective predictive utility.

Several limitations warrant consideration. First, the retrospective and single-center design introduces potential selection bias, and the relatively modest sample size (*n* = 56) increases the risk of overfitting in regression and ROC analyses, thereby limiting the stability and generalizability of the findings. Second, group assignment was based on the final clinical outcome of surgery versus non-surgical management; therefore, the present analyses demonstrate association and discriminatory performance within a retrospective dataset, but do not establish genuine preoperative predictive value. Third, although major clinical covariates were adjusted in multivariable models, the observed microbiota differences may still be influenced by disease severity, antibiotic exposure, feeding pattern, and other treatment-related factors. Fourth, although compositional-data-aware analyses were added in the revised manuscript, 16S rRNA sequencing still provides relative rather than absolute quantification, and taxonomic resolution at the species level remains limited. Accordingly, the main practical significance of this study is to provide candidate microbial markers and an analytic framework for future prospective, longitudinal, and multicenter validation, rather than to establish an immediate clinical decision tool. Future investigations should further explore associations among microbial metabolites (e.g., short-chain fatty acids), host genetic polymorphisms, and surgical outcomes.

In summary, this study systematically characterized the association between gut microbiota features and surgical intervention status in preterm infants with NEC. Several microbiota markers remained significantly associated with surgical NEC after adjustment for key clinical covariates, suggesting that microbial dysbiosis is linked to a more severe inflammatory phenotype and to surgical NEC beyond some major measured confounders, although residual confounding cannot be excluded. Because the present study was retrospective and outcome-defined, these findings should not be interpreted as establishing prospective predictive performance or direct guidance for surgical timing. Rather, they provide candidate microbial markers for future prospective, longitudinal, and multicenter validation studies.

## Data Availability

The data presented in the study are deposited in the NCBI Sequence Read Archive (SRA) under BioProject accession number PRJNA1471472, http://www.ncbi.nlm.nih.gov/bioproject/1471472.
