# Supplementary material for: Gut microbiota profiling for risk stratification of surgical intervention in preterm infants with necrotizing enterocolitis: a retrospective cohort study
Source: Front Med (Lausanne). 2026 Jun 1;13:1827128. doi: 10.3389/fmed.2026.1827128 (PMC13267481; doi:10.3389/fmed.2026.1827128)
Supplement: Supplementary file 1 [file Supplementary_file_1.docx]

Supplementary Table S1. ANCOM-BC validation results for differentially abundant taxa between non-surgical and surgical NEC groups

| **Taxon** | **Taxonomic level** | **Mean relative abundance in non-surgical group (%)** | **Mean relative abundance in surgical group (%)** | **log fold change (surgical vs non-surgical)** | **SE** | **W statistic** | **P value** | **FDR-adjusted q value** | **Direction in surgical group** |
| --- | --- | --- | --- | --- | --- | --- | --- | --- | --- |
| Firmicutes | Phylum | 19.08 | 12.61 | -0.81 | 0.23 | -3.52 | <0.001 | 0.004 | Decreased |
| Proteobacteria | Phylum | 52.49 | 86.37 | 1.16 | 0.25 | 4.64 | <0.001 | <0.001 | Increased |
| γ-Proteobacteria | Class* | 42.31 | 33.17 | -0.61 | 0.2 | -3.05 | 0.002 | 0.014 | Decreased |
| Clostridia | Class | 2.31 | 0.98 | -0.74 | 0.24 | -3.08 | 0.002 | 0.008 | Decreased |
| Bifidobacterium | Genus | 29.05 | 23.62 | -0.67 | 0.22 | -3.03 | 0.003 | 0.011 | Decreased |
| Lactobacillus | Genus | 22.36 | 17.6 | -0.56 | 0.21 | -2.67 | 0.008 | 0.018 | Decreased |
| Salmonella | Genus | 5.02 | 6.21 | 0.49 | 0.18 | 2.72 | 0.007 | 0.021 | Increased |
| Clostridium | Genus | 4.52 | 7.81 | 0.76 | 0.24 | 3.17 | 0.002 | 0.006 | Increased |
| Enterobacteriaceae | Family | 18.74 | 31.28 | 0.88 | 0.27 | 3.26 | 0.001 | 0.005 | Increased |
| Bacilli | Class | 16.85 | 11.42 | -0.53 | 0.19 | -2.79 | 0.005 | 0.017 | Decreased |

Abbreviations: ANCOM-BC, analysis of compositions of microbiomes with bias correction; SE, standard error; FDR, false discovery rate.
Note: Differential abundance analysis was performed between the non-surgical NEC group and the surgical NEC group using ANCOM-BC. Positive log fold change values indicate higher abundance in the surgical group, whereas negative values indicate lower abundance in the surgical group. Multiple testing was adjusted using the Benjamini–Hochberg procedure.
*γ-Proteobacteria was analyzed as a subgroup within Proteobacteria based on the taxonomic annotation used in the present study.


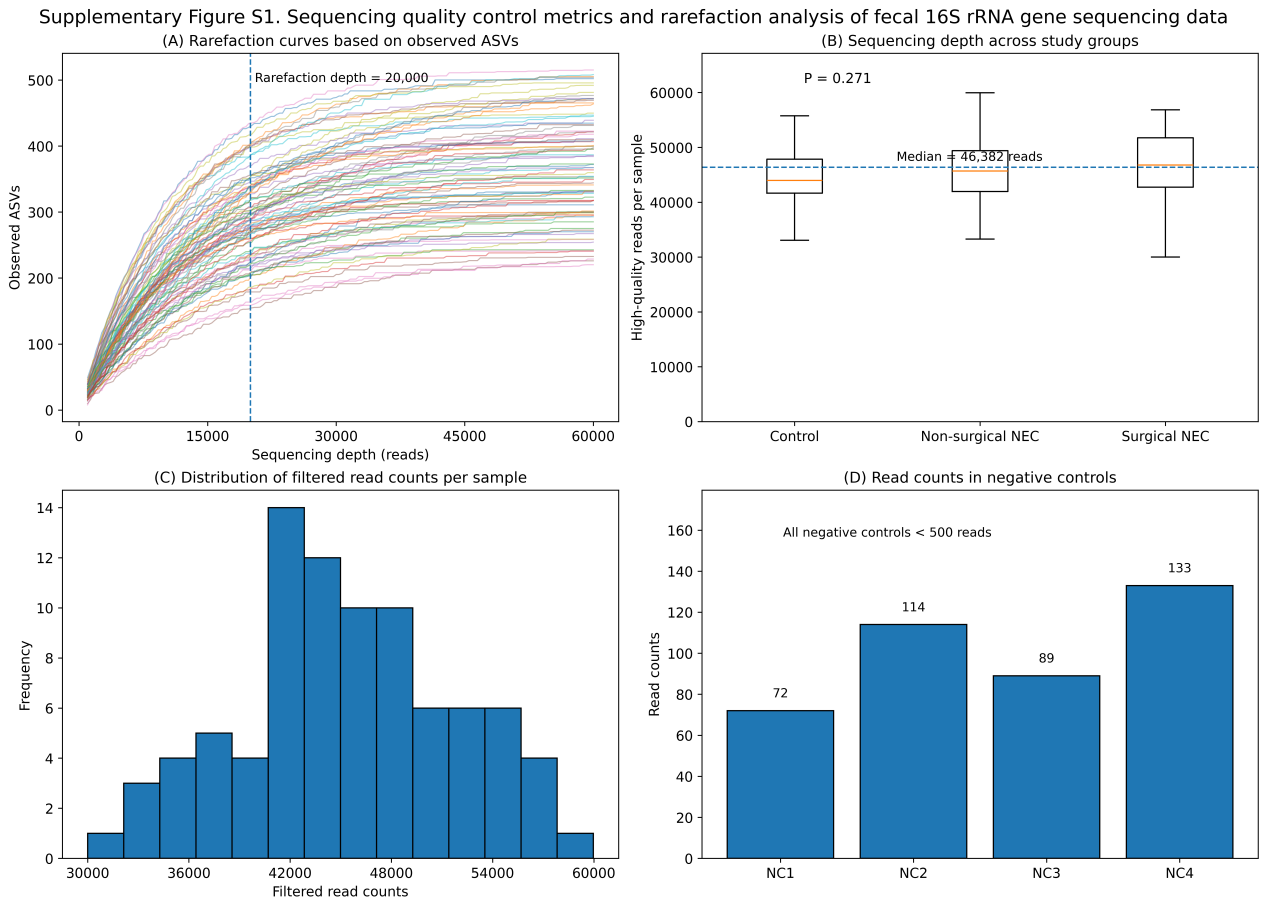


Supplementary Figure S1. Sequencing quality control metrics and rarefaction analysis of fecal 16S rRNA gene sequencing data.
(A) Rarefaction curves based on observed ASVs for all included fecal samples. Most curves approached a plateau with increasing sequencing depth, indicating adequate sampling of microbial diversity.
(B) Sequencing depth across the control, non-surgical NEC, and surgical NEC groups. The median number of high-quality reads per sample was 46,382 (interquartile range, 39,115–54,806), with no significant difference among groups (P = 0.271).
(C) Frequency distribution of filtered read counts per sample after denoising, chimera removal, and ASV filtering.
(D) Negative control samples yielded negligible read counts, supporting the absence of substantial experimental contamination.


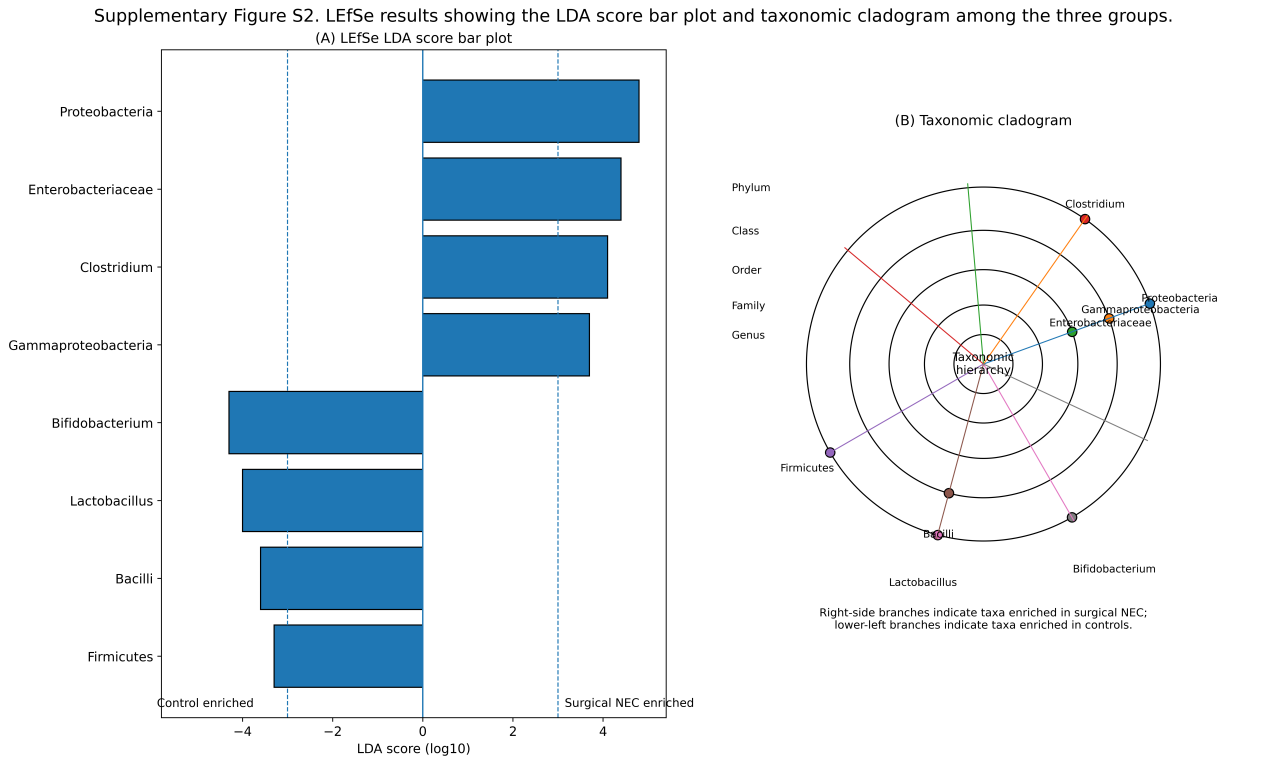


Supplementary Figure S2. LEfSe results showing the LDA score bar plot (A) and taxonomic cladogram (B) among the three groups.
